# Sclerosing Mesenteritis Presenting With Small Bowel Obstruction in a Patient With Systemic Lupus Erythematosus: A Case Report

**DOI:** 10.7759/cureus.61796

**Published:** 2024-06-06

**Authors:** Muzi Meng, Harsh R Parikh, AAM A Baqui, Daniel T Farkas

**Affiliations:** 1 Medicine, American University of the Caribbean School of Medicine, Cupecoy, SXM; 2 General Surgery, BronxCare Health System, New York, USA; 3 Medicine, St. George's University School of Medicine, St. George's, GRD; 4 Pathology, BronxCare Health System, New York, USA; 5 Surgery, BronxCare Health System, New York, USA

**Keywords:** systemic lupus erythematosus (sle), exploratory laparotomy, mesenteric fibrosis, small bowel obstruction, sclerosing mesenteritis

## Abstract

Sclerosing mesenteritis (SM) is a rare inflammatory disorder characterized by chronic inflammation and fibrosis of the mesenteric adipose tissue. While SM can manifest with various gastrointestinal symptoms, its association with small bowel obstruction (SBO) is infrequent. We present a case of a 78-year-old male with a history of systemic lupus erythematosus (SLE) who presented with acute abdominal pain and distention. The patient had multiple admissions with the same symptoms. A CT scan showed swirling of the proximal central mesentery, small bowel malrotation with volvulus, and high-grade mechanical obstruction of the proximal jejunum. The patient underwent exploratory laparotomy, with findings significant for multiple inflammatory nodules in the mesentery. These were causing adhesions between the bowel and mesentery, resulting in a volvulus of the bowel. One segment was resected, and subsequent histopathological examination revealed subserosal fibrosis and chronic inflammation. The clinical scenario was consistent with a diagnosis of SM. This case highlights the challenges of diagnosing and managing SBO in the presence of SM and SLE. Further research is needed to understand the underlying pathophysiological mechanisms and improve management techniques for this rare clinical condition.

## Introduction

Sclerosing mesenteritis (SM) is an uncommon inflammatory disorder marked by persistent inflammation and fibrosis in the mesenteric adipose tissue, resulting in thickening and distortion of the mesentery [1]. Although SM can present with a range of gastrointestinal symptoms, such as abdominal pain and diarrhea, its occurrence with small bowel obstruction (SBO) is extremely rare [2]. Furthermore, the co-occurrence of SM with systemic lupus erythematosus (SLE), an autoimmune disease marked by systemic inflammation and tissue damage, adds additional clinical complexity.

SBO, a severe complication of SM, involves the mechanical or functional blockage of the small intestine, typically resulting in abdominal pain, distention, nausea, vomiting, and potential ischemic complications [2]. The concurrent presence of SM and SLE, especially when accompanied by the complications of SBO, poses distinctive challenges in diagnosis and management [1]. The autoimmune dysregulation inherent in SLE may exacerbate the inflammatory process in SM, potentially increasing the likelihood of mesenteric fibrosis and subsequent bowel obstruction [3].

Due to the infrequent occurrence of this clinical presentation, there is a restricted comprehension of the optimal diagnostic approach and management strategies for SBO in individuals with simultaneous SM and SLE. This case report seeks to enhance the current literature by detailing a distinctive instance of SBO in a patient with SLE and concurrent SM, emphasizing the diagnostic challenges and management nuances within this intricate clinical context.

## Case presentation

The patient was a 78-year-old male who was presented to the emergency department with a one-day history of diffuse abdominal pain, rated a seven, with two episodes of non-bilious and non-blood vomiting. On review of symptoms, the patient also mentioned a one-day history of constipation, obstipation, and progressively worsening nausea. During the physical exam, palpation revealed mild abdominal distention with pain on deeper palpation. In a review of the patient's history, the patient revealed five prior admissions in the preceding three years for subacute intestinal obstruction. In a review of the previous admissions, the patient was treated with nasogastric tube decompression and improved each time without requiring surgery. Furthermore, they noted a remote history of bilateral inguinal hernia repair but did not recall the details of the procedure. The patient reported a history of SLE of over 40 years, managed with daily 10 mg prednisone under the supervision of an outside rheumatologist. The patient was admitted for conservative management of SBO via nasogastric decompression, as proven successful in previous admissions.

**Figure 1:**
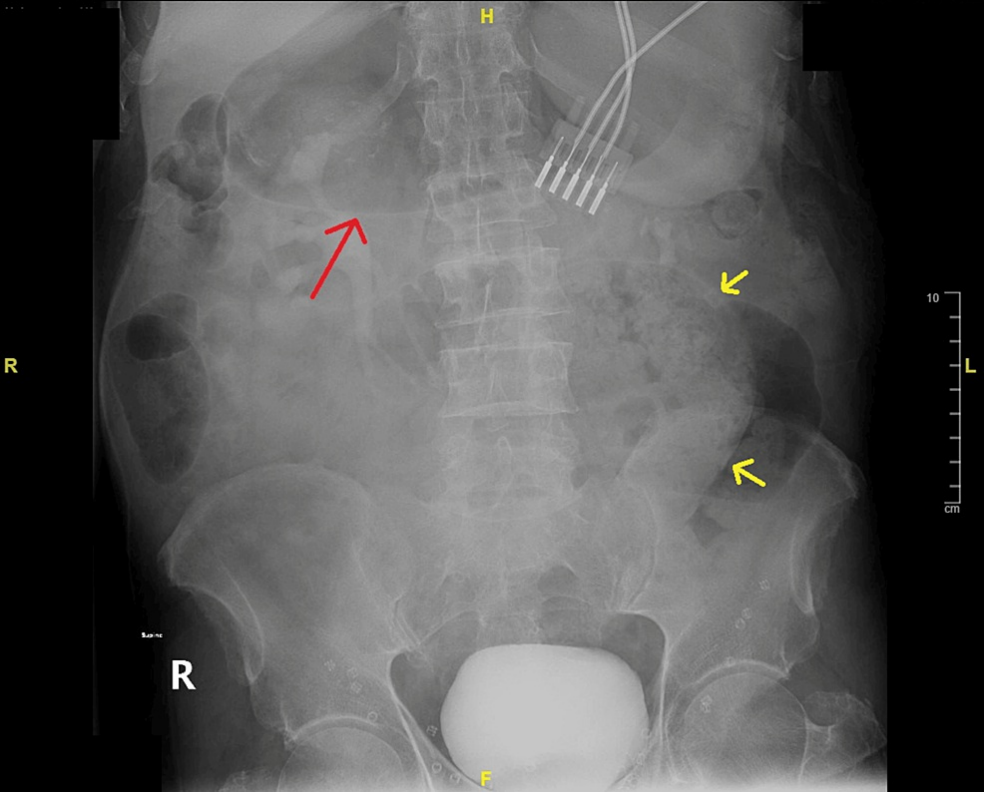
Upright abdominal X-ray showing a fecalized small bowel loop to the left of the spine (yellow arrows) with a distended stomach (red arrow)

**Figure 2:**
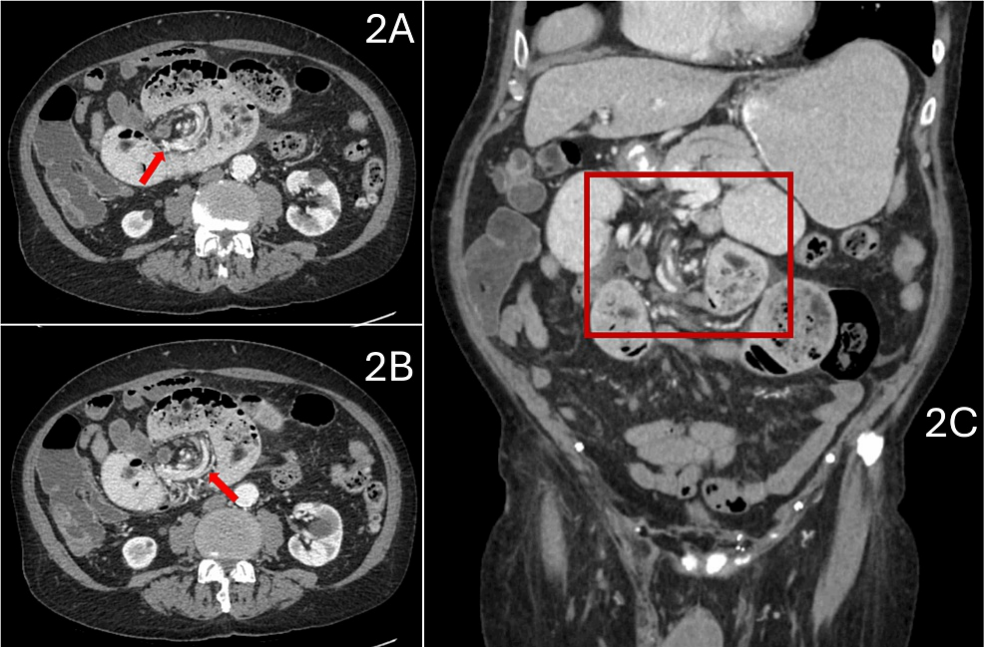
CT imaging of the abdomen and pelvis 1A and 1B: CT axial imaging of the abdomen/pelvis following oral contrast demonstrating whirring mesentery at the site of obstruction (red arrows). Significant distention of the proximal bowel loops can also be identified adjacent to the obstruction. This creates a fecalization sign that is suggestive of long-standing obstruction. 1C: CT coronal imaging of the abdomen/pelvis following oral contrast with the whirring of the mesentery at the site of the obstruction (red box). Noticeable distal collapse of bowel loops can also be identified to evidence the diagnosis of SBO. CT: computed tomography, SBO: small bowel obstruction

An abdominal X-ray was captured in the ED and presented a fecalized small bowel loop with a distended stomach (Figure 1). Further CT scans of the abdomen and pelvis showed swirling of the mesentery at the site of obstruction with proximal dilated loops and a collapsed distal bowel. Metal tacks were noted in bilateral groins on the CT, indicative of laparoscopic bilateral inguinal hernia repair. In this admission, the CT abdomen/pelvis with oral and IV contrast again showed whirling of the proximal central mesentery, small bowel dilatation, and high-grade mechanical obstruction of the proximal jejunum (Figure 2). Following admission, conservative measures were attempted to resolve the SBO, specifically via nasogastric decompression as done in previous admissions. Delayed films showed some contrast reaching the colon, but there were still signs of obstruction and dilation of the proximal bowel loops with collapsed distal loops. After discussion with the surgical team and counseling of the patient, the patient was planned for an exploratory laparotomy for definitive management of the currently diagnosed SBO.

**Figure 3:**
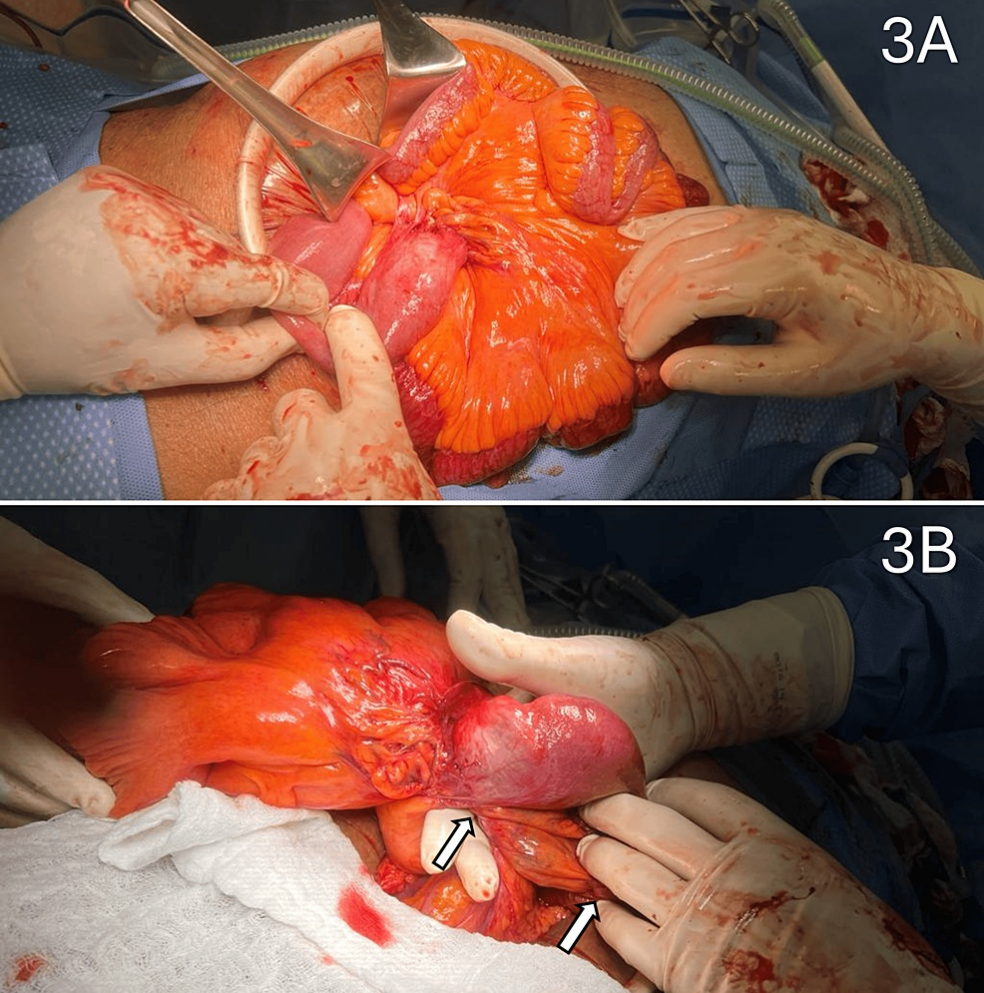
Mesentery shortening and bowel kinking 3A: Intraoperative photograph of the mesenteric shortening. 3B: Intraoperative photograph of the kinking of the bowel (white arrows)

During the surgery, multiple sites of adhesions were found throughout the mesentery. The serosa of the bowel was adherent to the mesentery in multiple locations. These adhesions created external obstructive forces, causing the entire mesentery to be significantly narrowed and twisted in consequence. This led to the volvulus of the proximal small bowel segments seen on the CT scan (Figure 3). Of note, there were no adhesions to the inguinal regions, and the mesh was well incorporated. The affected bowel was all in the upper abdomen. The adhesions to the inflammatory nodules on the mesentery were all divided. There was one area where there was a densely adherent adhesion in proximity to the proximal superior mesenteric artery. It could only be partially lysed due to the concern of injuring the artery and compromising circulation to the entire small bowel. As such, the remaining segment of the bowel remained narrowed due to the inflammatory adhesion. Approximately 12 cm of unhealthy dusky jejunum was resected, followed by a primary anastomosis. Surgical adhesiolysis and resection allowed for the resolution of the proximal small bowel volvulus. Post-operatively, rheumatology was consulted for the patient’s underlying SLE. Due to elevated anti-DNA antibodies and decreased C3 and C4 complement factors, the patient was suggested to restart their normally prescribed 10 mg prednisone with hydroxychloroquine in the event of a symptomatic flare. The post-operative recovery period was uneventful. The patient was kept on clear liquids till post-operative day 3 and discharged home on post-operative day 8. On discharge, the patient was tolerating a regular diet and having consistent bowel movements. The patient was followed in the outpatient clinic at regular intervals for two months post-operatively. All visits were unremarkable with no recurrent symptoms, and the patient reported feeling better than they had in years.

**Figure 4:**
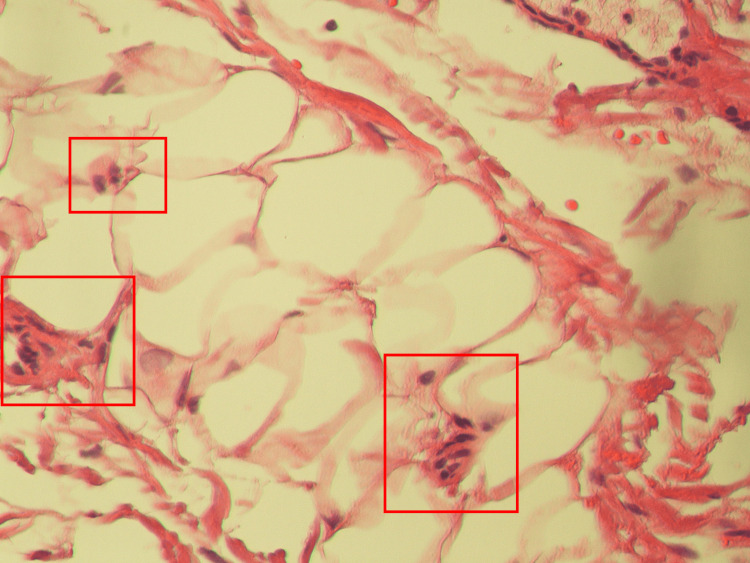
Histopathological slide of the mesenteric adipose tissue A slide for the dissected small segment of the small bowel was viewed at 1000x magnification. This slide demonstrates submucosal and subserosa congestion with signs of chronic inflammation (red boxes) and no signs of vasculitis. Together with the clinical picture, this provides evidence supporting the hypothesized diagnosis of SM. SM: sclerosing mesenteritis

The pathology on the small bowel segment showed submucosal and subserosal congestion with subserosal fibrosis and mild chronic inflammation (Figure 4). Histopathological findings demonstrated no evidence of vasculitis. Together with the clinical picture, this was consistent with a final diagnosis of SM.

## Discussion

SM is a rare inflammatory condition of the mesentery, characterized by chronic, non-specific inflammation, fibrosis, and fat necrosis [1]. Clinically, SM presents with abdominal pain and distension, nausea and vomiting, and mass effect symptoms [2,4]. The occurrence of SBO within the context of SM in a patient with SLE represents an uncommon but significant clinical scenario. The occurrence of SBO within an underlying diagnosis of SLE complicates the clinical course, presentation, and management. This involves either consideration for enhanced complication risk from immunosuppression as a product of SLE management or adjustment of the risk-benefit ratio in surgical vs. non-surgical management of SBO within the setting of underlying SLE [2-5]. Additionally, it is possible to oversee the connection between SBO and the underlying SLE, allowing for only the possibility of isolated symptomatic SBO management without adequate attention given to the inflammatory etiology. Therefore, prompt evaluation, identification, and clinical management adjustments are required for appropriate management and maximizing clinical outcomes. This case underscores the importance of considering uncommon etiologies in the presentation of SBO and their potential complications: ischemic compromise, associated autoimmune conditions, and intussusception [2-7], in the case of this patient, specifically involving the underlying setting of SLE.

The pathogenesis of SBO in SM is multifactorial. Specifically, mesenteritis involves the thickening of the abdominal mesentery from acute inflammatory reactions, either due to infection or autoimmune processes. Persistent and progressive inflammation of tissues and membranes can lead to consequent thickening, adhesion, fibrosis, and the formation of scar tissue. Recurrent inflammation and fibrosis of the mesentery can form transverse-extending fibrous bands, resulting in the constriction and compression of the segments of the small bowel, ultimately leading to mechanical luminal obstruction [5]. Additionally, mesenteric fibrosis can distort normal bowel anatomy, predisposing to volvulus or intussusception. This further contributes to SBO risk. Within the setting of SLE, the underlying autoimmune process can exacerbate the inflammatory cascade within the mesentery, intensifying the fibrotic changes and predisposing to SBO development [3]. Furthermore, the concurrent presence of vasculitis, a common manifestation of SLE, may induce ischemic changes within the mesenteric vessels, adding complexity to the clinical course [6]. However, histopathological imaging demonstrated no vasculitis in this case, only evidence for chronic inflammatory changes. The multifactorial interplay underscores the intricate mechanisms involved in the development of SBO in patients with SM, particularly in the context of autoimmune disorders like SLE. A proper understanding of these mechanisms is crucial for effective management and intervention strategies aimed at alleviating bowel obstruction and addressing the underlying autoimmune pathogenesis.

The diagnosis of SM can be challenging due to its nonspecific clinical presentation and lack of specific biomarkers. The diagnostic process embraces a holistic clinical approach, incorporating diverse elements, most notably imaging studies involving CT scans or MRIs. CT findings typically include a heterogenous mass with soft tissue attenuation, fat stranding, and occasional calcifications within the mesentery [5]. MRI may demonstrate a low-signal intensity mass on T1-weighted images and a high-signal intensity on T2-weighted images, reflecting the fibrous and inflammatory components of the lesion [7]. Lastly, histopathological evidence can include evidence of chronic inflammatory changes in the submucosa and serosa layers. SLE-associated vasculitis is also common in the histopathological diagnosis of SM. This is particularly visualized with the congestion of inflammatory cells in deeper tissue layers. However, these histopathological signs are usually non-specific and non-characteristic. Therefore, these findings have to be combined with the overall clinical picture to arrive at a final diagnosis of SM.

The concurrent management of SM and SLE requires a comprehensive multidisciplinary approach that addresses the complexities of both conditions while not distracting clinical focus from the presenting clinical diagnosis of SBO. Primary therapeutic strategies should be directed toward controlling the underlying autoimmune pathology inherent to SLE. This often involves the use of immunosuppressive agents such as corticosteroids and other disease-modifying antirheumatic drugs in the modulation of systemic autoimmune inflammatory processes characteristic of SLE [8,9]. However, the use of immunosuppressive agents within the setting of SM requires careful consideration due to concerns about exacerbating mesenteric inflammation; specific concerns involve inflammation and malignancy risk due to an associated immunocompromised state from chronic immunosuppressant dependence [8,10]. In this case, SLE was managed conservatively with corticosteroids, with the avoidance of targeted immunosuppression due to a history of recurrent lymphoma and associated malignancy. This represents the balancing act in conservative pharmacodynamic management for patients with SM and concurrent autoimmune disorders, such as SLE [8]. However, surgical intervention is considered in individuals who are unresponsive to conservative management and continue to exhibit signs of correlated complications, such as a mechanically obstructive SBO as exhibited in this case.

The diagnosis of SBO in a patient with SLE and SM requires a comprehensive approach, including clinical evaluation and imaging studies such as abdominal CT scans [10]. Conservative management is often the initial approach to managing SBO in this complex clinical scenario; this includes nasogastric decompression, bowel rest (nil per oral), or a Gastrografin challenge. In patients with significant obstruction, refractory obstruction, or complications such as bowel ischemia or perforation, prompt surgical intervention becomes imperative. Surgical options vary based on the severity and extent of obstruction, ranging from lysis of adhesions to segmental bowel resection [11]. This determination requires a meticulous evaluation of the patient's clinical status, encompassing the presence of refractory obstruction, the severity of the symptoms, and the likelihood of complications. Surgical intervention aims to alleviate obstruction, normalize bowel function, and minimize the potential for additional complications.

With various therapeutic options available to offer patients, continuous research strives to pinpoint the optimal strategy, seeking ongoing refinement and the development of improved modalities to enhance overall patient outcomes. The intricate nature of the condition underscores the crucial need for collaborative initiatives involving healthcare specialists, fostering a multidisciplinary approach that integrates the expertise of rheumatologists, gastroenterologists, and surgeons. This collaborative effort ensures a comprehensive and well-coordinated strategy, addressing diverse aspects of the patient's condition. The dynamic evolution of therapeutic interventions not only has the potential to alleviate symptoms but also advances long-term management strategies, ultimately contributing to an enhanced quality of life. As research advances, multidisciplinary collaboration between surgical and medical specialists can work to create innovative strategies to improve the management of SBO in the setting of SLE and SM. This involves a combination of medical and pharmaceutical management with communication and collaboration to weigh surgical options for care, enhancing effectiveness in navigating the complexities of this condition.

## Conclusions

SBO, in the presence of SLE and SM, presents a distinct and complex clinical scenario that necessitates a comprehensive and multidisciplinary approach to diagnosis and treatment. Timely detection of SBO is essential to reduce the risk of serious complications such as intestinal ischemia and perforation, which are linked to elevated morbidity and mortality. Our study demonstrates that surgical intervention with continued medical management can provide a successful outcome in the event of recurrent SBO due to an underlying autoimmune etiology. Further research can provide improved insights into the underlying pathophysiological mechanisms and polish treatment strategies to be specific to individuals with SM and SLE diagnoses. Continuous collaboration among rheumatologists, gastroenterologists, radiologists, and surgeons is imperative to optimize patient care and enhance outcomes within this intricate patient demographic.
